# MDM2 degraders for Merkel cell carcinoma: round peg in a round hole

**DOI:** 10.1172/JCI208535

**Published:** 2026-07-01

**Authors:** Sarki A. Abdulkadir

Merkel cell carcinoma (MCC) is a rare but highly aggressive skin cancer with poor outcome and limited therapeutic options (1). It arises from Merkel cells, a neuroendocrine cell type found in the top layer of the skin, and is commonly associated with clonal integration of the Merkel cell polyoma virus, also known as human polyomavirus 5 (2). The expression of polyoma virus small T antigen in MCC leads to activation of MDM2, which results in degradation of the tumor suppressor protein p53 (3). Accordingly, virus-positive MCCs rarely mutate the *TP53* gene, making them good candidates for strategies aimed at restoring functional p53 tumor suppressor protein as a therapeutic strategy.

MDM2 inhibitors that disrupt the interaction between MDM2 and p53 can lead to p53 stabilization in MCC with antitumor effects; however, the stabilized p53 protein induces MDM2 expression in a feedback loop, which is thought to limit the efficacy of such strategies (4, 5). Using higher doses of MDM2 inhibitors to achieve sustained inhibition is not feasible due to dose-limiting toxicities that result from activation of p53 in bone marrow and gastrointestinal cells (4). Therefore, strategies aimed at degrading MDM2 appear to be an attractive approach to disrupt the MDM2/p53 feedback loop and potentially result in enhanced antitumor efficacy.

Ongoing efforts have yielded a number of MDM2 degraders that are being tested for use in various malignancies (6). In this issue of the *JCI*, Ananthapadmanabhan et al. examined the efficacy of MDM2 degraders including a clinical stage PROTAC degrader, KMT-253, in models of MCC, with encouraging results (7). MDM degraders showed superior efficacy compared with inhibitors. With the aid of mathematical modeling, the authors showed that MDM2 degraders disrupt the p53/MDM2 feedback loop, unlike MDM2 inhibitors, explaining the higher potency of MDM2 degraders in stabilizing p53 protein. Furthermore, proteomics studies indicated that MDM2 degraders lead to increased protein levels of direct p53 targets, including the cell cycle inhibitor CDKN1A (p21).

The clinical significance of the work by Ananthapadmanabhan and colleagues lies in choosing a disease where an MDM2 degrader is likely to have maximum impact. A highly aggressive cancer with limited therapeutic options that frequently retains the WT *TP53* gene like MCC is a perfect candidate for emerging anti-MDM2 therapies. Nevertheless, the study by Ananthapadmanabhan et al. still leaves some unanswered questions. Although the MDM2 degraders appear to be superior to MDM2 inhibitors in MCC models, it is still not clear from the study if degraders, like inhibitors, will be encumbered by bone marrow toxicity, thereby limiting clinical utility.

## Conflict of interest

SAA is a cofounder and a share holder in Vortex Therapeutics and Degromics Bio.

## Version 1 07/01/2026

Electronic publication
